# Case report: *TTMV::RARA*-positive pediatric APL with spinal cord compression as initial presentation: unique clinical features and therapeutic outcomes revealed by a 12-case systematic cohort analysis

**DOI:** 10.3389/fonc.2026.1742593

**Published:** 2026-05-08

**Authors:** Hong-Lan Yang, Guo-Yan Xin, Mei-Ting Long, Dan- Chen, Xun Zhao, Meng-Jun Huang, Zheng-Lian Yao, Lin-Qiang Shen, Jian-Juan Ma, Yuan-Yuan Tuo, Yan- Li, Xiao-Yan Yang

**Affiliations:** Department of Pediatrics, The Affiliated Hospital of Guizhou Medical University, Guiyang, China

**Keywords:** acute promyelocytic leukemia, extramedullary involvement, pediatric, TTMV::RARA, venetoclax

## Abstract

This case report describes a 9-year-old boy with *TTMV::RARA*-positive acute promyelocytic leukemia (APL) presenting with spinal cord compression due to vertebral destruction, a manifestation not previously documented in the literature. Through a systematic review of 11 published cases (2020–2024), we identified both shared and distinctive features of this ultra-rare entity. The patient presented with a 2-year history of progressive hip pain, culminating in neurogenic claudication and urinary retention. Diagnostic evaluation revealed APL-like morphology and immunophenotype (CD33^+^, MPO^+^, CD34^−^, HLA-DR^−^), negative *PML::RARA* fluorescence *in situ* hybridization (FISH), and a high burden of *TTMV::RARA* fusion detected by RNA sequencing (81,142 copies). Treatment with all-trans retinoic acid (ATRA) plus an oral arsenic compound showed an insufficient response; however, the addition of venetoclax achieved molecular remission, which was maintained through 10 months of follow-up. Magnetic resonance imaging (MRI) documented resolution of spinal compression with fatty marrow replacement post-treatment. The literature review reveals recurring patterns: frequent extramedullary involvement (7/12 cases) and diagnostic challenges with conventional testing (6/10 cases FISH/reverse transcription-polymerase chain reaction (RT-PCR) negative). While limited by a single-case observation and heterogeneous reported data that preclude statistical analysis, this report expands the recognized clinical spectrum of *TTMV::RARA* APL and documents three previously unreported observations: spinal cord compression as the initial presentation, venetoclax-induced remission, and oral arsenic compound utilization. These findings suggest RNA-based fusion testing may be informative for *PML::RARA*-negative suspected APL with atypical presentations, although optimal diagnostic and therapeutic approaches await validation through collaborative studies.

## Introduction

Acute promyelocytic leukemia (APL), a distinct subtype of acute myeloid leukemia (AML) accounting for approximately 10–15% of AML cases, is most commonly driven by *PML::RARA* fusions (1). Emerging non-canonical *RARA* rearrangements, including *TTMV::RARA*, pose diagnostic and classification challenges (2). We present a pediatric *TTMV::RARA* APL case with an unusual presentation of spinal cord compression, a manifestation not previously reported in the 11 published cases identified in our literature review. RNA sequencing revealed a *TTMV::RARA* fusion with high transcript abundance, consistent with a cryptic, virus-associated rearrangement. The patient achieved molecular remission after induction therapy with all-trans retinoic acid (ATRA) plus Compound Huangdai Tablets (an arsenic-containing formulation); serial monitoring demonstrated that *TTMV* transcript levels correlated with treatment response. This case not only expands the clinical spectrum of *TTMV::RARA* APL but also highlights its potential for extramedullary involvement, warranting further investigation into virus-associated genomic events in leukemogenesis.

## Case description

A 9-year-10-month-old boy presented on December 20, 2024, with a 2-year history of progressively worsening bilateral lower limb pain (involving thighs and hips). Over the preceding year, the pain evolved to intermittent claudication, with nocturnal lumbar pain developing in the last 2 months. Twenty days before admission, he experienced acute urinary retention and markedly limited mobility, without preceding trauma or fever.

On examination, the patient was alert but lethargic and lay in a semi-reclined position. An indwelling urinary catheter was in place with scant pale-yellow urine output. Diffuse tenderness was noted over both lower extremities, hips, and lumbosacral region, with mild midline lumbar spinous processes tenderness. Neurological examination revealed asymmetric lower extremity weakness (right 3 of 5; left 2 of 5), limited by pain and neurogenic claudication) with a diminished left patellar reflex (1+ of 4). Superficial and deep sensation were preserved bilaterally. Cardiorespiratory examination was unremarkable.

Laboratory findings (Baseline): Complete blood count showed white blood cell count 8.36 × 10^9^/L, hemoglobin 116 g/L, and platelet count 337 × 10^9^/L. Coagulation studies revealed hypofibrinogenemia (0.93 g/L),elevated D-dimer (16.97 μg/mL), and increased fibrin/fibrinogen degradation products (57.80 μg/mL).

**Figure 1:**
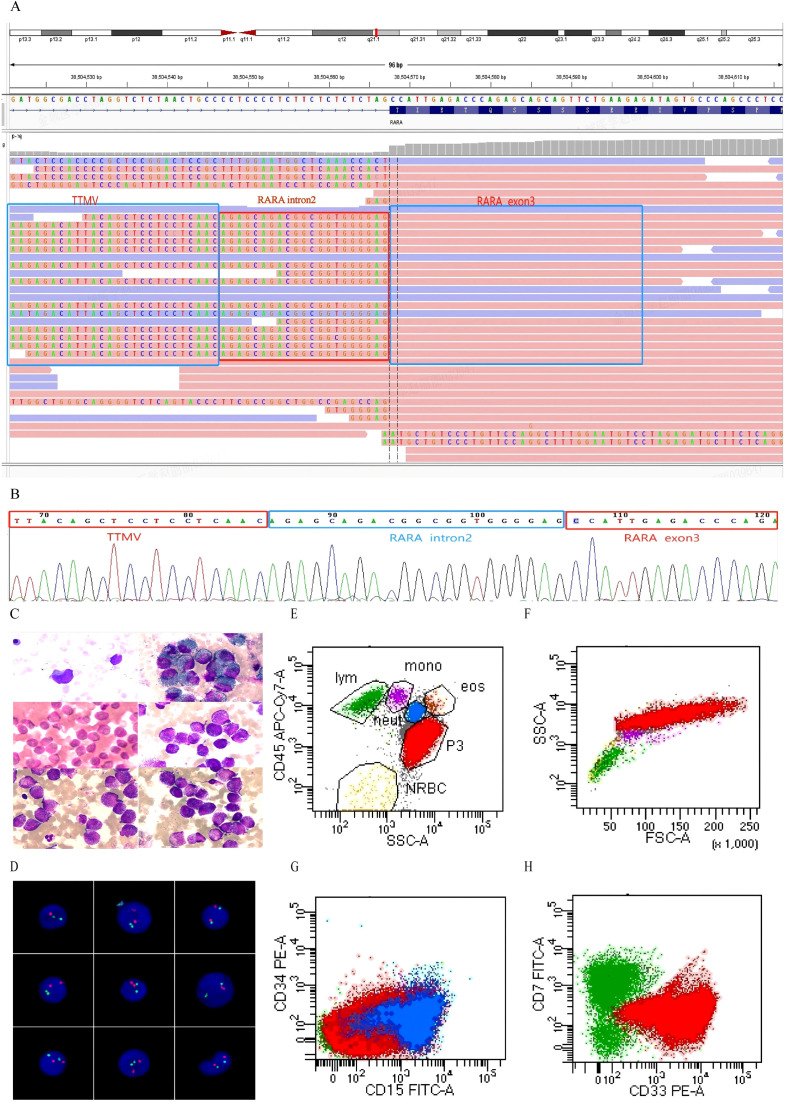
Clinical features and genetic analysis of the *TTMV::RARA* APL cases. **(A)** Integrative Genomics Viewer (IGV) visualization of RNA sequencing data showing TTMV insertion upstream of *RARA* exon 3. Soft-clipped reads at the breakpoint indicate the fusion junction, with boxed regions highlighting TTMV sequences, *RARA* intron 2, and *RARA* exon 3.**(B)** Sanger sequencing confirming two genomic junction sequences of the *TTMV::RARA* integration/fusion. **(C)** Bone marrow smear at initial presentation showing a predominance of abnormal promyelocytes. **(D)** Fluorescence *in situ* hybridization (FISH) analysis using LSI *PML/RARA* dual-color, dual-fusion probe showing absence of *PML::RARA* fusion. Normal signal pattern with 2 red *(RARA*) and 2 green (*PML*) signals observed in representative interphase nuclei (0/200 cells positive).**(E–H)** Flow cytometry immunophenotyping of bone marrow aspirate: **(E)** Identification of abnormal myeloid population (red, 88.1% of nucleated cells) with CD45dim expression, distinct from lymphocytes (green, 3.4%) and monocytes (purple, 0.9%).**(F)** Forward scatter (FSC) versus side scatter (SSC) analysis demonstrating increased cell size and granularity characteristic of abnormal promyelocytes.**(G)** Absence of both CD15 and CD34 expression in the abnormal population, showing the typical CD34 negative APL immunophenotype.**(H)** Strong expression of myeloid marker CD33 with absence of aberrant CD7 expression, confirming myeloid lineage without lymphoid marker co-expression.

Diagnostic workup (MICM): Morphology/Cytochemistry: Bone marrow aspiration demonstrated 95% hypergranular promyelocytes (**Figure 1C**). Cytochemical staining showed strong myeloperoxidase (MPO/POX) positivity (100%) and diffuse periodic acid-Schiff (PAS) positivity (100%). Immunophenotyping: Flow cytometry of bone marrow identified an abnormal myeloid population (88.1%) with CD45^dim^ expression and an APL-like phenotype: CD33^+^, MPO^+^, CD13^+^, CD9^+^, CD123^+^, CD117^partial+^, and CD64^dim^, with absence of CD34 and HLA-DR; lymphoid markers were negative (CD7^--^, CD19^--^) (**Figures 1E–H**). Cytogenetics/Molecular: FISH using an LSI *PML/RARA* dual-color, dual-fusion probe was negative for *PML::RARA* (0/200 nuclei; 0.00%; laboratory cutoff 7%) (**Figure 1D**). RNA sequencing was performed using total RNA extracted from bone marrow (TRIzol method, RIN 8.2) with 1 μg input for library preparation (NEBNext^®^ Ultra™ Kit). Sequencing on Illumina NovaSeq X Plus platform (paired-end 150 bp, 25 Gb data) achieved 92% alignment rate to hg19 using STAR aligner. The targeted 318-gene RNA panel detected a high-abundance *TTMV::RARA* fusion (81,142 copies; *TTMV::RARA/ABL1* ratio 28.33%), with a viral-host junction consistent with *TTMV* integration into *RARA* intron 2 upstream of exon 3 (**Figure 1A**). Comprehensive mutation screening via RNA-seq including *FLT3* (*ITD/TKD*), *NPM1*, *NRAS*, *KRAS*, *WT1*, *CEBPA*, *RUNX1*, *ASXL1*, and *TET2* revealed no pathogenic variants (minimum 5% VAF, 500× depth). The fusion junction was further confirmed by Sanger sequencing (**Figure 1B**). Paired tumor/normal whole-exome sequencing identified no additional reportable pathogenic/likely pathogenic alterations; three germline variants of uncertain significance were reported (*ZBTB16* p.I264N, *FCGR3A* p.R36S, and *HYOU1* p.K816N) (**Table 1**).

**Table 1:** Clinical and molecular characteristics, frontline therapy, and outcomes of *TTMV::RARA* acute promyelocytic leukemia cases.

Patient (Ref)	Age/Sex	Initial presentation	Extramedullary disease (site, any time)	Morphology/Immunophenotype	Genetics (fusion & mutations)	Frontline therapy	Status at last follow-up
Pt. 1(Astolfi,et al.2021^[18]^)	6/F	Fever, bone pain, bruising/bleeding	NA	APL-like; APL phenotype	*TTMV::RARA.*	7+3+ATRA → CR → allo-HSCT	Alive, in remission after HSCT
Pt. 2(Astolfi, et al.2021^[18]^)	3/NA	NR	NA	NA	*TTMV::RARA*	NA	NA
Pt. 3(Sala-Torra,et al, 2022^[19]^)	39/M	Bleeding, thrombocytopenia; DIC-like labs	NA	APL-like; APL phenotype	*TTMV::RARA.*	7+3+ATRA (IF) → DNR/Ara-C/VP-16+VEN (CR)	Alive
Pt. 4(Xue Chen,et al 2022^[20]^)	3/M	Persistent fever, headache	Relapse: multiple metastases	APL-like; APL phenotype (CD34 partial+)	*TTMV::RARA; FLT3-ITD*	ATRA+HU → DNR/Ara-C/VP-16 (CR) → allo-HSCT	Relapsed post-HSCT (50 days); treatment withdrawn
Pt. 5(Chen, Jiaqi, et al,2023^[21]^)	11/F	Pelvic pain, intermittent fever	NA	APL-like	*TTMV::RARA*; *RARA* c.1160T>C (p.I387T); *ARID1B* c.2837_2838insGT (p.A947Sfs*4).	ATRA+IDA (CR) → allo-HSCT	Alive,CR post- HSCT
Pt. 6(Linya Wang, et al,2024^[22]^)	9/M	Abdominal pain, fever.	CNS/meninges at diagnosis; T3 vertebra/intervertebral foramen	APL-like; APL phenotype (CD34 low)	*TTMV::RARA*; *ARID1A* c.4524T > A/p.Y1508* LBD c.826C > G/p. R276G c.860C > G/p.S287W c.1061C > G/p.P354R	VEN-based therapy → allo-HSCT (5 months post-diagnosis)	Relapsed; awaiting 2nd allo-HSCT
Pt. 7(Zhao Wang, et al ,2024^[23]^)	15/M	Fatigue, intermittent fever	Relapse : CNS/intracranial; CSF blasts+	APL-like; APL phenotype	*TTMV::RARA*; *NRAS* c.35G>A (p.G12D); *TCF3* c.265_278del (p.S89Pfs*11)	ATRA+HHT+Ara-C (IF)	Refractory (no CR)
Pt. 8(Harrison K,et al,2024^[24]^)	11~20/M	Hearing loss, ear fullness	Myeloid sarcoma (temporal bone/maxilla/sphenoid); CNS	APL-like; APL phenotype	*TTMV::RARA*; *NRAS* c.35G>T (p.G12V)	Ara-C+DNR+VP-16	Died,PD
Pt. 9(Harrison K,et al,2024^[24]^)	0~5/F	Hip pain, decreased activity	CNS (CSF+)	APL-like; APL phenotype (HLA-DR low)	*TTMV::RARA; WT1* c.1142C>A (p.S381*); *TRRAP* p.S722F.	Induction chemotherapy + ATRA → HiDAC+VP-16+ATRA → HSCT	Alive,CR post-HSCT
Pt. 10(Harrison K,et al,2024^[24]^)	0~5/M	Fever, weight loss, leg/hip pain; coagulopathy	NA	APL-like; APL phenotype	*TTMV::RARA*	Ara-C+DNR+VP-16 → HSCT	Alive,CR
Pt. 11(Harrison K, et al,2024^[24]^)	21~30/F	Leg pain; distal femur mass	Myeloid sarcoma (femur/LN/kidneys)	APL-like; APL phenotype	*TTMV::RARA*; *KMT2C* p.Q2635*; *WT1* p.R369*; *ATR* c.6320-2A>C.	7+3 (CR) → HSCT	Alive,CR
Pt. 12(present case)	9/M	Chronic thigh/hip pain → neurogenic claudication; spinal cord compression	Spine/epidural involvement causing compression (presenting feature)	APL-like; APL phenotype	*TTMV::RARA.*	ATRA+RIF → +VEN → CR	Alive,CR

7+3, cytarabine plus an anthracycline-based induction regimen; allo-HSCT, allogeneic hematopoietic stem cell transplantation; APL, acute promyelocytic leukemia; APL-like, acute promyelocytic leukemia-like; Ara-C, cytarabine; ATO, arsenic trioxide; ATRA, all-trans retinoic acid; AZA, azacitidine; CNS, central nervous system; CR, complete remission; CSF, cerebrospinal fluid; DIC, disseminated intravascular coagulation; DNR, daunorubicin; F, female; FLT3-ITD, FMS-like tyrosine kinase 3 internal tandem duplication; HHT, homoharringtonine; HLA-DR, human leukocyte antigen-DR isotype; HSCT, hematopoietic stem cell transplantation; HU, hydroxyurea; IDA, idarubicin; IF, induction failure; LN, lymph node(s); LDAC, low-dose cytarabine; M, male; MP, 6-mercaptopurine; NA, not available; ND, not done; NR, not reported; PD, progressive disease; RIF, Realgar-Indigo naturalis formula (Huangdai tablet); VEN, venetoclax; VP-16, etoposide. For selected cases, age is shown as an interval (e.g., 11-20 years) because an exact age was not reported in the original publication.

**Figure 2:**
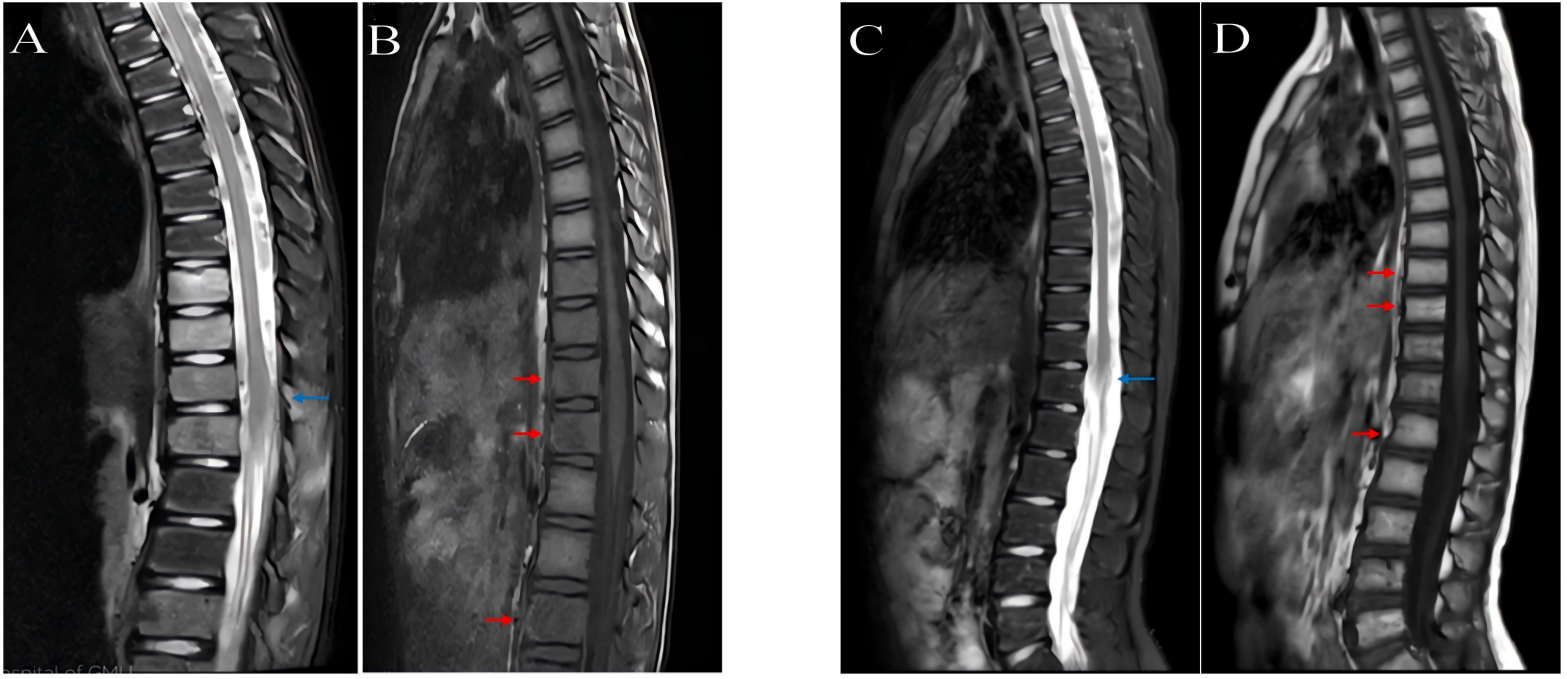
Spinal MRI findings in a 9-year-old boy with *TTMV::RARA* APL. **(A)** Sagittal T2-weighted image shows severe spinal cord compression at the T10–11 level (blue arrow) with mild intramedullary T2 hyperintensity, consistent with compressive edema, and severe spinal canal narrowing. **(B)** Sagittal T1-weighted image demonstrates diffuse low T1 marrow signal in the T10 (upper arrow), T12 (middle arrow), and L3 (lower arrow) vertebral bodies with loss of normal fatty marrow signal, consistent with marrow involvement. **(C)** Sagittal T2-weighted image at the 10-month post-treatment follow-up shows marked improvement of spinal canal narrowing at T10–11 and normalization of spinal cord signal (blue arrowhead), with resolution of compressive edema. **(D)** Sagittal T1-weighted image at follow-up shows interval fatty marrow reconstitution in the T10, T12, and L3 vertebral bodies (red arrowheads), with patchy hyperintensity replacing the pretreatment diffuse hypointense marrow pattern.

Imaging and other studies: Computed tomography (CT) and magnetic resonance imaging (MRI) revealed multiple osteolytic lesions with associated soft-tissue masses involving T10-L5 vertebrae and the pelvis (**Figures 2A, B**), resulting in spinal canal stenosis and cord compression. PET/CT demonstrated mild heterogeneous uptake in the involved bones and reactive lymphadenopathy in the cervical and axillary regions. Electrocardiography showed normal sinus rhythm.

Diagnosis: Based on MICM findings, including bone marrow morphology consistent with promyelocytic leukemia with hypergranular promyelocytes (95%), an APL-like immunophenotype (CD45^dim^, CD33^+^, MPO^+^, CD34^-^, with variable CD117), negative *PML::RARA* by FISH, and detection of a high-abundance *TTMV::RARA* fusion by targeted RNA sequencing (81,142 copies; *TTMV::RARA/ABL1* ratio 28.33%), the patient was diagnosed with acute promyelocytic leukemia with *TTMV::RARA* fusion, complicated by extramedullary osseous/paravertebral involvement, spinal cord compression, and coagulopathy.

Treatment course and outcomes: Induction therapy with ATRA (25 mg/m²/day) plus Compound Huangdai Tablets (0.135 g/kg/day) was administered for 2 months, but complete remission (CR) was not achieved. Venetoclax (100–150 mg/m²/day for 7 days) was subsequently added, resulting in morphologic CR. Consolidation consisted of three cycles: (1) ATRA + Huangdai + venetoclax with intrathecal chemotherapy; (2) addition of high-dose cytarabine (1 g/m²/day for 2 days); and (3) addition of idarubicin (10 mg/m²/day for 2 days). Maintenance therapy was continued for 72 weeks, alternating Cycle A (Huangdai, ATRA, methotrexate, and 6-mercaptopurine) and Cycle B (ATRA, methotrexate, and 6-mercaptopurine), with venetoclax 100 mg once daily throughout maintenance. Clinically, bone pain improved within 1 week and resolved by 1 month; spinal mobility began to improve from week 2, and the catheter was removed after 2 weeks. Coagulation parameters stabilized within 2 weeks. The *TTMV::RARA/ABL1* transcript became undetectable after 2 months of therapy and remained negative at the most recent follow-up (February 28, 2025). Follow-up MRI at 10 months showed resolution of T10–11 spinal canal stenosis on sagittal T2-weighted images (**Figure 2C**), and T1-weighted images demonstrated fatty marrow replacement in the T10, T12, and L3 vertebral bodies compared with the pretreatment diffuse hypointense infiltration pattern (**Figure 2D**).

## Discussion

*TTMV::RARA* APL represents an exceptionally rare subtype of *RARA*-rearranged leukemia. Although prior reports have described its clinical phenotype, molecular features, and treatment outcomes in considerable detail (3, 4), the limited number of published cases means that the phenotypic spectrum remains to be more clearly delineated, and substantial interpatient heterogeneity is evident. Here, by reporting our new case (Pt.12) and systematically comparing it with 11 previously reported cases (18–24), we sought to define recurrent clinical and laboratory features and to identify divergent clues that may hinder early recognition and timely diagnosis and/or suggest an inadequate response to differentiation therapy with *ATRA* with or without arsenic.

### Shared features across published cases and the present patient

Across reported *TTMV::RARA* APL cases, the presenting morphology and immunophenotype are largely APL-like and may therefore channel patients into management pathways designed for canonica*l PML::RARA* APL. Our comparison with previously published cases (**Table 1**) is purely descriptive and aims to summarize recurrent patterns. Among cases with available flow cytometry data (10/12), myeloid-lineage markers were commonly reported (CD33 positive in 9/10; MPO positive in 6/10), while HLA-DR and CD34 were usually absent or low (HLA-DR negative/low in 8/10; CD34 negative/low in 8/10), with occasional exceptions (e.g., partial CD34 expression), highlighting phenotypic heterogeneity (**Supplementary Table 1**). Our case aligns with this overall profile (CD33^+^, CD13^+^, MPO^+^, CD45^dim^, CD34^−)^ but showed only dim CD64, which is notable because strong CD64 is often emphasized in classical *PML::RARA* APL (5, 6). Collectively, these observations suggest that morphology and immunophenotyping alone may be insufficient to distinguish *TTMV::RARA* APL from canonical *PML::RARA* APL or other *RARA*-rearranged entities; thus, when APL is strongly suspected but routine *PML::RARA* testing is negative, *RNA*-based fusion testing should be pursued to identify cryptic *RARA* rearrangements such as *TTMV::RARA (*7).

Aggregation of reported cases reveals that the defining molecular event in *TTMV::RARA* APL is *TTMV* insertion near *RARA* intron 2/exon 3, generating fusion transcripts with variable junction sites (**Supplementary Table 2**). Unlike the relatively clustered breakpoints in *PML::RARA (*8), *TTMV* insertion sites within *RARA* intron 2 vary between patients (e.g., Pt. 1: chr17:40334196; Pt. 4: chr17:40332265), producing fusion isoforms retaining different lengths of *RARA* intronic sequences (**Supplementary Table 2**). Limited descriptive data suggest that cases retaining longer intronic fragments may achieve molecular remission more slowly (e.g.Pt. 1: 38 nt vs Pt. 3: 14 nt; **Supplementary Table 2**), though this observation requires validation in larger cohorts. Given this molecular heterogeneity, our case’s complete remission following venetoclax addition to *ATRA*/arsenic suggests that for patients with suboptimal early response to differentiation therapy (**Supplementary Table 3**), venetoclax may be considered as an anti-apoptotic targeting strategy under individualized assessment. The absence of *FLT3* and *NPM1* mutations in our case provides supplementary evidence that the fusion event alone may drive treatment-responsive survival programs in some patients, though causal inference remains premature at this stage.

Integrated analysis of 12 *TTMV* integration cases reveals non-random insertion patterns adjacent to *RARA* intron 2/exon 3, suggesting dual mechanisms involving “integration-favorable chromatin landscapes” and “post-integration clonal selection.” (9).

From a chromatin accessibility perspective, *RARA* expression peaks during promyelocyte differentiation, with active transcription creating an open chromatin state that significantly increases the probability of exogenous nucleic acid insertion (10). This phenomenon aligns with the preferential integration of HTLV-1 and HIV into transcriptionally active regions (11–13). The high fusion transcript burden detected in our cohort (81,142 copies/μg RNA, representing 28.33% of *ABL1*), combined with Wang et al.’s ATAC-seq data demonstrating overlap between *TTMV* insertion sites and DNase I hypersensitive regions in *RARA* intron 2 (14), collectively supports the “transcription-dependent chromatin accessibility” framework.

Regarding functional selection, despite variable breakpoints within intron 2, all cases in our series consistently preserve the *RARA* ligand-binding domain (LBD, exons 4-9) while disrupting the N-terminal regulatory region. This structural convergence parallels classical *PML::RARA* fusion, where the LBD not only participates in leukemogenesis but also determines ATRA sensitivity (15, 16). The therapeutic response to ATRA plus arsenic trioxide (ATO) induction therapy observed in our cohort (with subsequent complete remission achieved through venetoclax combination) confirms the retained pharmacological modulability of the retinoid signaling pathway. In contrast, *PLZF::RARA* frequently exhibits ATRA resistance due to altered corepressor complex interactions (17), suggesting that different fusion partners shape therapeutic response heterogeneity through specific domain retention patterns.

Extramedullary involvement has been documented in multiple *TTMV::RARA* APL cases, including CNS disease, myeloid sarcomas, and in our patient (17), destructive vertebral lesions with spinal cord compression—the latter representing a previously unreported manifestation. These extramedullary presentations, whether at diagnosis or relapse (**Table 1**), pose a shared clinical challenge of potentially directing patients to non-hematology specialties initially. Our patient’s 2-year diagnostic odyssey through orthopedic and neurology departments before hematologic evaluation exemplifies this diagnostic complexity. This pattern across cases underscores the importance of considering *TTMV::RARA* APL in pediatric patients presenting with unexplained bone lesions or CNS involvement, particularly when accompanied by cytopenias.

### Descriptive features of the present case (Pt.12)

While sharing APL-like morphology with published cases(**Figure 1C**), our patient presented with distinctive clinical features warranting documentation. The 2-year history of progressive hip pain evolving to neurogenic claudication and ultimately spinal cord compression represents a previously unreported presentation timeline. Although bone pain and extramedullary involvement have been documented in prior cases, the vertebral destruction pattern (T10-L5) with neurological compromise (**Figures 2A, B**) and subsequent fatty marrow replacement post-treatment has not been described (**Figures 2C, D**). This observation, while representing a single data point, suggests that *TTMV::RARA* APL may present along a broader clinical spectrum than currently recognized. For clinical practice, this case highlights the importance of considering hematologic evaluation in children with persistent bone/spinal symptoms and atypical promyelocytes.

Regarding therapeutic response, our case adds to the documented heterogeneity in treatment outcomes. The insufficient initial response to ATRA plus oral arsenic compound followed by complete remission after venetoclax addition represents one treatment experience among varied approaches reported. While no efficacy claims can be made from a single case, this observation aligns with emerging literature on venetoclax combinations in challenging clinical scenarios. Rather than proposing treatment recommendations, we document this experience as part of the accumulating clinical record for this ultra-rare entity.

### Limitations and future directions

#### Limitations

This single-case report has inherent limitations. Observations from one patient cannot establish treatment efficacy or disease patterns. The aggregated 12 cases from different centers preclude statistical analysis. Our 10-month follow-up is insufficient for long-term outcome assessment, and optimal treatment strategies remain undefined without prospective validation.

#### Future directions

This case documents three previously unreported observations: spinal cord compression presentation, venetoclax-induced molecular remission, and oral arsenic compound use. Based on these findings and literature review, we suggest considerations for future case documentation:

1. Diagnostic approach: RNA-based fusion testing may be informative for *PML::RARA*-negative suspected APL with extramedullary features, though optimal testing algorithms await validation.
2. Clinical documentation: Systematic recording of treatment sequences, response timelines, and MRD kinetics when available would facilitate pattern recognition across cases.
3. Collaborative efforts: Given the ultra-rare nature of *TTMV::RARA* APL, multicenter data collection using standardized parameters (extramedullary sites, coagulation profiles, treatment responses) may help identify clinically relevant patterns over time.

These suggestions represent observational insights rather than evidence-based recommendations, awaiting validation through larger collaborative studies.

## Data Availability

The datasets presented in this study can be found in online repositories. The names of the repository/repositories and accession number(s) can be found in the article/**Supplementary Material**.
