# Supplementary material for: Case report: TTMV::RARA-positive pediatric APL with spinal cord compression as initial presentation: unique clinical features and therapeutic outcomes revealed by a 12-case systematic cohort analysis
Source: Front Oncol. 2026 May 8;16:1742593. doi: 10.3389/fonc.2026.1742593 (PMC13194065; doi:10.3389/fonc.2026.1742593)
Supplement: Supplementary file 1 [file Table1.docx]

**Table S1 Baseline clinical, laboratory, and molecular features of *TTMV::RARA* patients**

| **Patient  number** | **Morphology** | **Immunophenotype** | **Initial Karyotype** | **Genetics**  **(fusion & mutations)** | **Initial WBC × 10^9^/L** | **Initial HGB g/L** | **Fibrinogen**  **g/L** | **D-dimer**  **µg/mL** | **PT**  **s** | **aPTT**  **s** |
| --- | --- | --- | --- | --- | --- | --- | --- | --- | --- | --- |
| Pt. 1^[18]^ | Abnormal promyelocytes with numerous azurophilic granules | CD33^+^, CD13^+^, CD38^+^, CD99^+^ HLA-Dr^low^ | NR | NR | 2.8 | 97 | ↓ | ↑ | ↑ | ↑ |
| Pt. 2^[18]^ | NR | NR | NR | NR | NR | NR | NR | NR | NR | NR |
| Pt. 3^[19]^ | Abnormal promyelocytes | CD33^+^ , MPO^+^  CD34^−^, HLA-DR^−^ | 46,XY,i(17)(q10)[18]/47,XY,+18,i(17)(q10)[2] | NR | NR | NR | ↓ | ↑ | ↑ | NR |
| Pt. 4^[20]^ | hyper-granular promyelocytes | CD13^+^, CD33^+^, CD45^+^, CD117^+^,  CD123 Partial^+^, CD64^+^, MPO^+^；CD34^+^, HLA-DR^−^, CD11b^−^, CD16^−^. | NR | *FLT3-ITD* | 36.76 | 86 | NR | NR | ↑ | ↑ |
| Pt. 5^[21]^ | hyper-granular promyelocytes | NR | 47,XX,+21[10] | *RARA* c.1160T>C/p.I387T； *ARID1B* c.2837_2838insGT:p.A947Sfs*4： | NR | NR | NR | NR | NR | NR |
| Pt. 6^[22]^ | aberrant promyelocytes  with abundant granules | CD13^+^, CD33^+^, CD38^+^, CD123^+^, MPO^+^, CD371^+^； CD34^low^, HLA-DR^−^ | 46,XY,i(17)(q10)[3]/47,XY,idem,+8[9]/46,XY,idem,add(16)(q24)[8] | *ARID1A* c.4524T > A/p.Y1508* *RARA* c.826C>G (p.R276G)、c.860C>G (p*.*S287W)、c.1061C>G *(*p.P354R) | 41.9 | 86 | NR | NR | NR | NR |
| Pt. 7^[23]^ | aberrant promyelocytes | CD13^+^, CD33^+^ HLA-DR^−^, CD34^−^, CD117^−^ | NR | *NRAS* c.35G>A/p.*G12D*； *TCF3* c.265_278del/p.S89Pfs*11 | 40.8 | 109 | ↓ | NR | ↑ | ↑ |
| Pt. 8^[24]^ | aberrant promyelocytes | CD43^+^, MPO^+^, CD34^−^, CD117^−^ , CD56 ^−^ | 46,XY,add(7)(q22)[5]/46,XY[15] | *NRAS* c.35G>T (p.G12V) | NR | NR | NR | NR | NR | NR |
| Pt. 9^[24]^ | azurophilic granules No Auer rods | CD13^+^, CD33^+^, CD38^+^, CD64^+^, CD34^−^, HLA-Dr ^low^ | NR | *WT1:* c.1142C>A *(*p.Ser381Ter) ;T*RRAP*: c.2165C>T (p.Ser722Phe) ;  deletion of *WT1* due to del(11)(p13);Partial gain of 17q, specifically dup(17)(q21.31q25.3) | 17 | NR | NR | NR | ↑ | NR |
| Pt. 10^[24]^ | hypergranular promyelocytic cells  with occasional Auer rods | MPO^+^, CD33^+^, CD117 Partial^+^, CD13^low^， HLA-DR^−^, CD34^−^. | 46,XY,der(7)ins(7;8)(q22;q13q22) | NR | NR | NR | NR | ↑ | ↑ | NR |
| Pt. 11^[24]^ | aberrant promyelocytes | CD33^+^, CD117 Partial^+^ , CD38 Partial^+^, CD13 Partial^+^, CD34^−^, HLA-DR^−^, CD15^−^, CD14^−^, CD56^−^, CD10^−^. | NR | *KMT2C* p*.*Q2635*, *WT1* p.R369*; *ATR* c.6320-2A>C | NR | NR | NR | NR | NR | NR |
| Pt. 12 | highly granulated promyelocytes,  filled with abundant azurophilic granules | CD33^+^, MPO^+^, CD13^+^, CD123^+^, CD45^dim^, CD64^dim^, CD9^+^, CD117 Partial^+^, CD19^−^, CD34^−^. | ND | *TTMV::RARA* (RNA-seq+; 81,142 copies; ratio 28.33%); no additional somatic P/LP variants (paired WES). | 8.36 | 116 | 0.93↓ | 16.97↑ | 10 | 31 |

**Table S2 Genetic characteristics of *TTMV::RARA* patients**

| **Patient  number** | **Methods for  fusion gene detection** | **Composition of the *TTMV::RARA* fusion gene** | **Genomic insertion site** | **Insert size of the fusion gene** | **Predicted  coding protein** |
| --- | --- | --- | --- | --- | --- |
| Pt. 1 | WTS | 38 nt of retained *RARA* intron 2 + 209 nt upstream of *RARA* exon 3 + *RARA* exon 3 | chr17:40334196 | 1045 bp | 485 |
| Pt. 2 | WTS | 45 nt of retained *RARA* intron 2 + 328 nt upstream of *RARA* exon 3 + *RARA* exon 3 | chr17:40333779 | NR | NR |
| Pt. 3 | Targeted NGS (*RARA* locus) | 14 nt of retained *RARA* intron 2 + *RARA* exon 3 | chr17:40338090 | 2400 bp | NR |
| Pt. 4 | WTS+WGS | 58 nt of *TTMV* UTR + 256 nt of *TTMV* ORF2 + *RAR*A exon 3 | chr17:40332265 | 1164 bp | 488 |
| Pt. 5 | WTS | *TTMV* ORF2 + 27 bp of *RARA* intron 2 + *RARA* DBD/LBD (splicing noted in original report) | *RARA* exon 3 5’ end, spliced to *RARA* intron 2. | NR | NR |
| Pt. 6 | WTS | *TTMV* ORF2 + 13 derived aa + 40 bp of *RARA* intron 2 + *RARA* exon 3 | Inserted at 5′ end of *RARA* exon 3; spliced to *RARA* intron 2 | NR | 462 |
| Pt. 7 | WTS | *TTMV* ORF2 + *RARA* intron 2 + *RARA* exon 3 | *TTMV* ORF2 sequence inserted into *RARA* gene intron 2 | NR | 478 |
| Pt. 8 | RNA-based | *TTMV* (MN768572.1):*:RARA* (exon 3–4) | *TTMV* ORF2, *RARA* intron 2 | 413 bp | NR |
| Pt. 9 | RNA-based & DNA-based | *TTMV* (MN770942.1)*::RARA* (intron 2; exon 3–4) | *TTMV* ORF2, *RARA* intron 2 | NR | NR |
| Pt. 10 | DNA-based | *TTMV* (MN771618.1)*::RARA* | *TTMV* ORF2, *RARA* intron 2 | NR | NR |
| Pt. 11 | DNA-based | *TTMV* (MN771921.1)*::RARA* | *TTMV* ORF1, *RARA* intron 2 | NR | NR |
| Pt. 12 | WTS | *TTMV* (MN770942.1)*::RARA* (intron 2; exon 3) | Inserted at 5′ end of *RARA* exon 3; spliced to *RARA* intron 2 | 21 bp | NR |

**Table S3 Treatment and outcome of *TTMV::RARA* patients**

| **Patient  number** | **Initial Therapy** | **Time to  complete  remission** | **Induction failure/Relapse** | **Relapse clinical features** | **Post-relapse  karyotype** | **Secondary therapy** |
| --- | --- | --- | --- | --- | --- | --- |
| Pt. 1 | Induction: Standard AML regimen (7+3) plus ATRA  Consolidation: HiDAC × 3 cycles | after the first induction cycle | Eight months after complete remission | fever, hemorrhagic findings, abdominal，muscular pain. | NR | 2 courses of ATO and ATRA |
| Pt. 2 | NR | NR | NR | NR | NR | NR |
| Pt. 3 | Standard 7+3+Modified DA+MIT+VP-16 +Ara-C + DAC+VEN | Induction failure, day 15 | Induction failure | NR | No | No |
| Pt. 4 | Initial: ATRA+HU (7d)  Induction: DNR+Ara-C+VP-16  Consolidation therapy: IDA, Ara-C, and VP-16 and achieved CR. | NR | one month after achieving CR | recurrent fever | NR | induction chemotherapy  and achieved remission by day 39 |
| Pt. 5 | Induction: ATRA + IDA  Consolidation: 4 standard cycles; Maintenance: 2 cycles  Regimen: ATRA + ATO + IDA  Response: Morphologic CR on day 38, followed by ATRA + Ara-C after 1 month  Salvage: ATRA + DNR + Ara-C × 1 cycle, persistent blasts | after the first induction cycle | stopped 12 months after the diagnosis. Relapse: 3 months after stopping the treatment. One more month later, BM morphological evaluation showed 46% aberrant promyelocytes | pelvic pain | 47,XX,+21[8]/48, XX,+8,+21[1]/46, XX[11] | decitabine, homoharringtonine, idarubicin, and cytarabine; evaluated 20 days after this regimen. BM morphology showed 11% aberrant promyelocytes, modified: ATRA, azacitidine, venetoclax, remission was achieved 13 days after the treatment |
| Pt. 6 | Induction regimens:  VEN (200mg/m²/d, d1-28) + LDAC (100mg/m²/d, d1-7)  Alternative: VEN (200mg/m²/d, d1-27) + AZA (75mg/m²/d, d1-7)  Consolidation: HHT (3mg/m²/d, d1-7) + Ara-C (1g/m²/d, d1-3)  CNS prophylaxis: Triple IT × 5 (MTX 10mg, Ara-C 50mg, DEX 5mg)  Definitive therapy: Allo-HSCT from father at 5 months post-diagnosis | NR | 13 months after allo-HSCT | thrombocytopenia PET-CT showed high-density shadows in the T3 vertebral body and intervertebral foramen(extramedullary infiltration.) | 47,XY, t(1;11) (p13; p11.2),  del(15) (q11.2q21),add(16) (q24), i(17) (q10),+21[20] | One cycle of ATRA (20 mg, days 1-28) , venetoclax (200 mg/m2, days 1-28) .  Ten days later,the second cycle of ATRA (25 mg, days 1-14),venetoclax (200 mg/m2, days 1-28), azacitidine (75 mg/m2, days 1-5), BM investigation revealed 65.5% blast cells; ATRA (20 mg, days 1-14), arsenic trioxide (0.15mg/kg, days 1-14). the blast cells in BM increased to 77.5% |
| Pt. 7 | ATRA (40 mg/d, d1-14), HHT (3 mg/d, d1-7), Ara-C (150 mg/d, d1-7)  Three courses of consolidation therapy;  ATRA (40 mg/d, d1-14),  ATO (10 mg/d, d1-28) | after the first induction cycle | one month post the last consolidation cycle | lower limb bone pain headache,  fever MRI revealed multiple intracranial lesions | 46,XY,t (3；20)(q26.2;q11[5]/46,idem,del (9)(p21[13]/46,XY[2]) | Relapse treatment: ATRA (40mg/d×14d) + IDA (12mg/d×3d) + Ara-C (200mg/d×7d) + IT (Ara-C+MTX); Consolidation: VEN (400mg×7d) + HHT (3mg/d×7d) + Ara-C (20mg bid×7d) + G-CSF (300μg/d×7d) × 2; 2nd relapse at 2 months; Salvage: Ara-C (150mg/d × 7d) + DNR (60mg/d × 3d) + VP-16 (150mg/d × 3d), failed CR |
| Pt. 8 | Standard dose Ara-C, DNR, VP-16;  Weekly IT therapy | NR | Three months  after initial diagnosis(relapse #1) | NR | NR | Relapse #1: HiDAC + mitoxantrone + thoracic RT → Bu/Cy conditioning → HSCT；Relapse #2 (7 months): Extramedullary (scalp MS, lymphadenopathy, skin lesions) → Liposomal DNR + FLAG → remission → 2nd HSCT |
| Pt. 9 | One course of standard dose induction chemotherapy, ATRA (d8-d21);  Consolidation therapy: HiDAC and VP-16 plus ATRA; HSCT | NR | NR | NR | NR | NR |
| Pt. 10 | Ara-C, DNR, VP-16; second induction with Ara-C and MIT; HSCT | NR | NR | NR | NR | NR |
| Pt. 11 | Induction: 7+3 (DNR+Ara-C) → CR; Consolidation: HiDAC; HSCT | NR | NR | NR | NR | NR |
| Pt. 12 | Induction: ATRA + RIF (2 mo) with inadequate response → add VEN (1 wk) → CR Consolidation: C1: ATRA + RIF + VEN+ IT (MTX + Ara-C + DEX); C2: C1 + HD Ara-C (1 g/m² ×2d). C3: C2 + IDA.Maintenance: Alternating cycles (72w): ① RIF + ATRA + MTX (20 mg/m²/w) + 6-MP (50 mg/m²/d); ② ATRA + MTX + 6-MP; ③ VEN (100 mg/d). | 2 months | No relapse at last follow-up | No | No | No |

**Abbreviations:** aPTT, activated partial thromboplastin time; AML, acute myeloid leukemia; Ara-C, cytarabine; ATO, arsenic trioxide; ATRA, all-trans retinoic acid; AZA, azacitidine; Bu/Cy, busulfan/cyclophosphamide; CNS, central nervous system; CR, complete remission; DEX, dexamethasone; DNR, daunorubicin; FLAG, fludarabine/cytarabine/G-CSF; G-CSF, granulocyte colony-stimulating factor; HiDAC, high-dose cytarabine; HHT, homoharringtonine; HSCT, hematopoietic stem cell transplantation; HU, hydroxyurea; IDA, idarubicin; IT, intrathecal; MIT, mitoxantrone; mo, months; MP, 6-mercaptopurine; MRI, magnetic resonance imaging; MTX, methotrexate; NGS, next-generation sequencing; ORF, open reading frame; PET-CT, positron emission tomography–computed tomography; PT, prothrombin time; RIF, Realgar–Indigo naturalis formula (Compound Huangdai Tablets); RT, radiotherapy; UTR, untranslated region; VEN, venetoclax; VP-16, etoposide; WGS, whole-genome sequencing; WTS, whole-transcriptome sequencing. NR,not reported;ND, Not done; No, as of last follow-up.Symbols: ↑, above the upper limit of normal; ↓, below the lower limit of normal.
